# Cariprazine’s efficacy in treating affective symptoms – pooled data from schizophrenia and bipolar depression trials

**DOI:** 10.1192/j.eurpsy.2022.419

**Published:** 2022-09-01

**Authors:** R. Mcintyre, Z. Dombi, Á. Barabássy, G. Németh

**Affiliations:** 1 University of Toronto, Department Of Psychiatry And Pharmacotherapy, Toronto, Canada; 2 Gedeon Richter Plc, Medical Division, Budapest, Hungary

**Keywords:** cariprazine, bipolar depression, schizophrénia

## Abstract

**Introduction:**

Affective symptoms are a common feature of schizophrenia and define bipolar disorder. Alterations in dopamine neurotransmission and activity at D_3_-D_2_ receptors is associated with depressive symptoms providing the rationale for targeting D_3_-D_2_ receptors with partial agonists.

**Objectives:**

The aim of the analysis herein is to examine and compare the efficacy of cariprazine in treating affective symptoms in both schizophrenia and bipolar depression.

**Methods:**

Data from 3 schizophrenia [NCT00694707, NCT01104766, NCT01104779] and 3 bipolar I depression studies [NCT013896447, NCT02670538, NCT0267055] were pooled for the analyses. To investigate efficacy across individual affective symptoms, the Marder anxiety/depression and negative symptom items of the Positive and Negative Syndrome Scale (PANSS) and single items of the Montgomery-Asberg Depression Rating Scale (MADRS) were analysed. Improvement across affective symptoms was examined primarily evaluating least square mean differences (LSMDs) in comparison to placebo in mean change from baseline.

**Results:**

The pooled ITT population was comprised of persons with schizophrenia (placebo=442, cariprazine=1024) and bipolar disorder (placebo=460, cariprazine=923). Cariprazine resulted in a significantly greater reduction when compared to placebo in three out of the four Marder anxiety/depression items; anxiety (p<0.01), tension (p<0.001) and depression (p<0.05). Similarly, cariprazine was significantly better than placebo in 9 out of the 10 MADRS individual items; apparent sadness (p<0.001), reported sadness (p<0.001), reduced sleep (p<0.05), reduced appetite (p<0.001), concentration difficulties (p<0.001), lassitude (p<0.001), inability to feel (p<0.001), pessimistic thoughts (p<0.01) and suicidal thoughts (p<0.05).

**Conclusions:**

The results herein indicate that cariprazine treatment is significantly effective at treating affective symptoms in persons with both schizophrenia and bipolar I depression.

**Disclosure:**

I am an employee of Gedeon Richter Plc.

